# Security in Intelligent Transport Systems for Smart Cities: From Theory to Practice

**DOI:** 10.3390/s16060879

**Published:** 2016-06-15

**Authors:** Muhammad Awais Javed, Elyes Ben Hamida, Wassim Znaidi

**Affiliations:** Qatar Mobility Innovations Center (QMIC), Qatar Science and Technology Park (QSTP), PO Box 210531, Doha, Qatar; elyesb@qmic.com (E.B.H.); wassimz@qmic.com (W.Z.)

**Keywords:** Intelligent Transport Systems, smart cities, Security and Privacy, Elliptic Curve Cryptography, quality of service, safety awareness, experimental benchmark, network simulations

## Abstract

Connecting vehicles securely and reliably is pivotal to the implementation of next generation ITS applications of smart cities. With continuously growing security threats, vehicles could be exposed to a number of service attacks that could put their safety at stake. To address this concern, both US and European ITS standards have selected Elliptic Curve Cryptography (ECC) algorithms to secure vehicular communications. However, there is still a lack of benchmarking studies on existing security standards in real-world settings. In this paper, we first analyze the security architecture of the ETSI ITS standard. We then implement the ECC based digital signature and encryption procedures using an experimental test-bed and conduct an extensive benchmark study to assess their performance which depends on factors such as payload size, processor speed and security levels. Using network simulation models, we further evaluate the impact of standard compliant security procedures in dense and realistic smart cities scenarios. Obtained results suggest that existing security solutions directly impact the achieved quality of service (QoS) and safety awareness of vehicular applications, in terms of increased packet inter-arrival delays, packet and cryptographic losses, and reduced safety awareness in safety applications. Finally, we summarize the insights gained from the simulation results and discuss open research challenges for efficient working of security in ITS applications of smart cities.

## 1. Introduction

**Figure 1:**
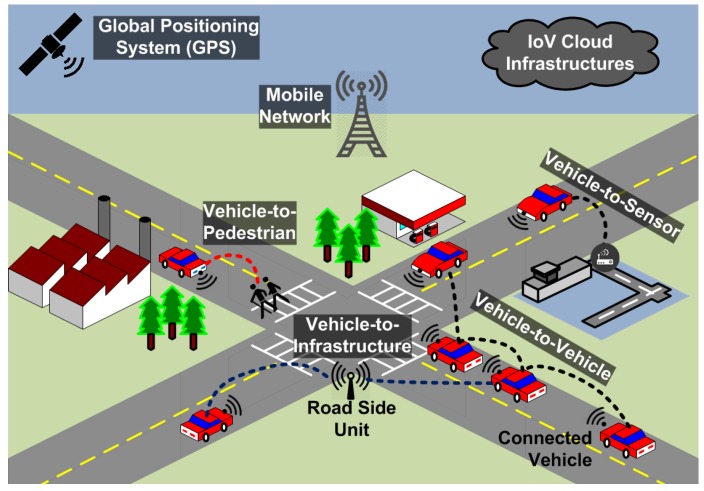
Smart cities communications architecture.

Intelligent Transport Systems (ITS) is the future technology that is envisaged to improve the road safety, traffic management and driver comfort. The concept behind such a system is ubiquitous connectivity of vehicles forming an Internet of Vehicles (IoV), hence providing them with a clear vision of traffic on the road [1,2,3]. Along with vehicle to vehicle connectivity, data is collected from infrastructure road side units (RSUs) and various other sensors placed on roads, buildings and human bodies constituting a *smart city* paradigm as shown in Figure 1. The collected data is disseminated to vehicle cloud that helps in managing the city level traffic, ensuring efficient warning notifications in terms of an emergency, providing vehicle’s wireless transceiver important information for congestion control and selection of security algorithms, and also taking steps to analyze the data quality and accuracy [4,5,6].

There are many applications in smart cities based on ITS including cooperative awareness, safe lane changing, safe intersection crossing, optimal traffic signal control, emergency warning notifications, smart parking allocation and downloading multimedia from internet [7,8,9,10]. Communications in ITS relies on two main modes namely vehicle to vehicle (V2V) and vehicle to infrastructure (V2I) as shown in Figure 1. Based on this wireless exchange of data, vehicles could maintain an improved neighborhood vision and safety.

Security is a key challenge in the implementation of ITS applications. There exist a plethora of security attacks today that can negatively impact the reliability of ITS applications. Particularly, the ITS security requirements include availability, authenticity, confidentiality, integrity and non-repudiation of the traffic and mobility data. Denial of service (DoS), jamming, broadcast tampering, man in the middle, Sybil, eavesdropping and message tampering are few of the common attacks that pose threat to the safety of vehicles in ITS [11,12,13].

To realize the large scale implementation of smart cities, it is critical for vehicles and other ITS stations to find secure solutions to combat the above mentioned attacks. Fortunately, ITS standards in US and Europe that have matured over the years now provide concrete security architecture and algorithms. The use of Elliptic Curve Cryptography (ECC) based algorithms have been proposed for digital signatures and encryption of ITS messages in these standards. Specifically, for digital signature, Elliptic Curve Digital Signature Algorithm (ECDSA) is used whereas Elliptic Curve Integrated Encryption Scheme (ECIES) with Advanced Encryption Standard (AES) is the default encryption algorithm [14,15].

While the security improves the reliability of applications in ITS and provides defense against various threats, it comes with a computation cost. This involves an increased packet size and delay required for signing and/or encrypting packets at the transmitter and verifying and/or decrypting them at the receiver. A higher packet size increases the end-to-end delay of safety packets and wireless channel occupancy. This could cause congestion particularly in high density traffic scenarios [16]. Another implication of using security in ITS is the loss of safety information. Generally, a vehicle will receive hundreds of packets per second and security processing (verification and decryption) all of them at the receiver will increase their packet delay. Due to stringent latency requirements imposed on safety packets, this results in reduced network QoS that translates directly to a lower vehicle safety in ITS [17]. Therefore, security and QoS have a trade-off and balancing each of these factors is an integral component of ITS.

**Contributions:** Security is considered as one of the most important milestone to enable the emergence of future smart cities and ITS applications. However, there is still a lack of comprehensive studies on the benchmarking of existing security standards in real-world settings, and on the evaluation of their impact on the achieved communications quality of service (QoS) and applications safety. Such studies can provide general guidelines for the design of future ITS and smart cities systems. The contributions of this paper are many-fold. First, we analyze and describe the implementation of the ETSI ITS security standard in conformance with the latest technical specifications (ETSI TS 103 097 [15]), including the security headers, certificate format, security profiles and ECC based algorithms. Second, using an extensive experimental study, we benchmark the performance of the implemented ETSI ITS security layer in terms of messages encapsulation (ENCAP) delays, messages decapsulation (DECAP) delays and messages security overheads, using various data payload sizes, hardware architectures and security algorithms. We also provide an online tool that presents our detailed ITS standard compliant security benchmark results that can be used for future research studies [18]. To the best of the authors knowledge such comprehensive study has not been conducted in the ITS context. Third, since experiment studies are limited to a few ITS nodes, we developed detailed NS-3 and SUMO simulation models based on the experimental results to evaluate the impact of security on the performance of vehicular communications in large scale urban/city scenario. With the help of simulation studies, we show the impact of security on Quality of Service (QoS) and safety awareness in the context of ITS applications. Finally, we derive the lessons learned and discuss further open research challenges in the domain of secure ITS communications.

The paper is organized as follows. Section 2 explains the key ITS enabling standards of smart cities, security threats and their countermeasures and existing work in the area of benchmark and evaluation of ITS security. Section 3 explains the standard based ITS security architecture in detail. We present the test-bed setup and experimental benchmark results in Section 4. This is followed by a simulation based study of smart city ITS security, its impact on QoS and future research challenges in Section 5. Finally, conclusions are drawn in Section 6.

## 2. Related Works

In this section, we discuss the existing work in the field of ITS applications for smart cities. The first subsection discusses the available standards in the field of ITS. The second subsection describes the different types of threats to the ITS applications and possible cryptographic solutions. In the third subsection, we explore the current literature related to benchmarking security procedures in ITS. In the final subsection, we discuss performance evaluation metrics for ITS.

### 2.1. Key Enabling Standards

Smart cities communications architecture is shown in Figure 1. Vehicles form the core component of ITS along with infrastructure road side units and traffic command center (TCC). Using Vehicle to Vehicle (V2V) and Vehicle to Infrastructure (V2I) communications, important traffic and mobility information is shared between different components of ITS. There currently exist two main ITS standards known as Wireless Access for Vehicular Environments (WAVE) [19] and European Telecommunications Standards Institute (ETSI ITS) [20] in United States and Europe respectively. Both of these standards define the complete network layered architecture including the MAC/PHY layer functions, transport and network layer mechanism, data traffic at the application layer, and security and management procedures for the ITS applications.

Since ITS applications depend on wireless exchange of information, specific data messages have been defined in both WAVE and ETSI ITS standards. Vehicles periodically broadcast their traffic and mobility information with each other and these messages are known as Basic Safety Messages (BSMs) in the WAVE standard and Cooperative Awareness Messages (CAMs) in the ETSI standard. Moreover, the ETSI standard defines a special message for disseminating warning notifications called Decentralized Environmental Notification Messages (DENMs) [12].

**Figure 2:**
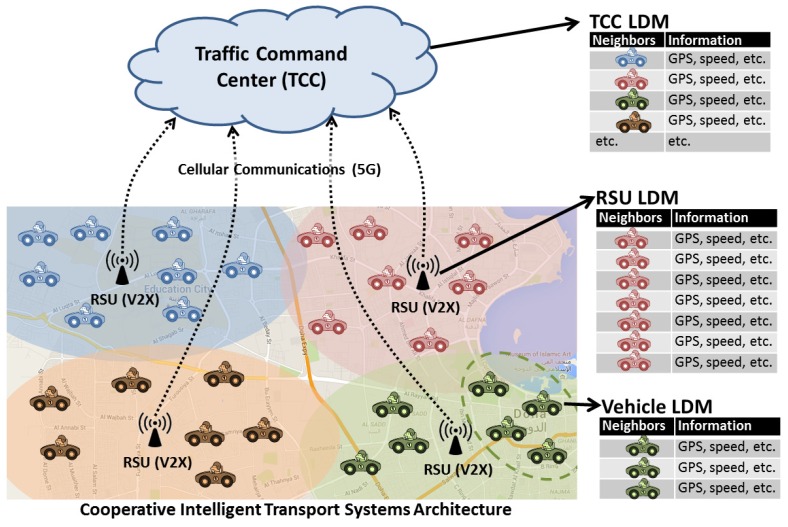
ITS based local dynamic map.

Using the information received in CAMs, each ITS station develops a local dynamic map (LDM) as shown in Figure 2 which is a database of neighborhood traffic [21]. With the help of LDM, vehicles could take critical driving decisions, RSUs could disseminate geographical warning notifications and TCC could manage city level traffic. The accuracy of LDM thus holds the key to the precision of traffic awareness of vehicles.

### 2.2. Security Threats and Countermeasures

Like other wireless networks, ITS is also vulnerable to various security attacks and appropriate countermeasures are required to secure the involved applications. The key requirements of a secure vehicular network includes availability that guarantees transmission of data within latency requirements using low overhead and light weight cryptographic algorithms. Confidentiality makes vehicle identities and data completely anonymous. Authentication is another key security feature that ensures messages are sent by a legitimate ITS station, the surrounding traffic locations are correctly verified and data attacks from malicious users are prevented. To define proper data access control for different ITS stations, authorization is a vital security requirement. Moreover, data integrity and validation that the data has not been tempered by a malicious user is another security challenge.

The list of security attacks, the security requirement they pose threat to, and the possible countermeasures are shown in Table 1. Denial of Service (DoS) attacks impact the availability service and in turn the QoS of safety applications. The attacks in this category include the jamming attacks that transmit noise signal on the physical channel to increase interference levels and distort communications. On the other hand, flooding attacks insert large amount of fake messages in the network to make the channel busy and unavailable. Sybil attacks adopt false node identities to transmit the dummy messages which can cause network congestion as well as disseminate false information in the network. Malware, Spamming, Black hole, Grey hole, Sink hole and Warm hole are few additional attacks that target the network availability. To overcome most of these attacks, digital signature algorithms can be used.

To target user confidentiality, eavesdropping and data interception are attacks that let malicious users access the sensitive information in the transmitted messages. Encryption is an effective cryptographic solution against such attacks. Vehicle authentication and authorization is compromised by attacks such as falsified entities, cryptographic replication, Global Navigation Satellite System (GNSS) spoofing, and timing attacks. In falsified entities attack, a user obtains a valid network identifier of an ITS station to transmit false messages. Cryptographic replication attacks create dummy certificates and keys to create ambiguity at the receiver during verification process. GNSS spoofing and timing attacks inject false location and timing of channel switching in the network to disrupt working of ITS. To overcome these attacks, encryption is required for critical messages with sensitive information.

Data Integrity is impacted by attacks such as Masquerading in which a vehicle uses a valid network identifier to broadcast itself as an emergency vehicle and thus effects movement of other vehicles. Another type of attack known as Data Playback attack in which malicious vehicles rebroadcast old messages within the network to generate false vehicle positions. Similarly, data alteration attacks can modify, delete or change the message content to create ambiguity in the network. Digital signature with certificate is a robust mechanism to minimize threats due to these attacks.

### 2.3. Evaluation of ITS Standard Compliant Security Architectures

ECC based algorithms are the default security schema in the ITS standard [19,20], including ECDSA and ECIES. Several past works have benchmarked the signature and verification procedures of ECDSA algorithm on different processors. The eBACS ECRYPT project [22] calculates signature and verification times as 1.88 ms and 2.2 ms respectively on an Intel core 2 Duo at 2.9 GHz. These values increase to 2.9 ms and 3.4 ms on an Intel Atom 330 at 1.6 GHz. In [23], ECDSA-NISTP256 security scheme for HTTPS application performance is evaluated. The signature time is 0.61 ms on an Intel i7 processor running at 3.4 GHz whereas the verification time is not mentioned in the paper.

Another benchmark implemented on Altera Cyclone II FPGA for ECDSA algorithm with key length of 163 bit requires 0.94 ms for signature generation and 1.61 ms for verification [24]. The experiment in [25] is conducted using a Xilinx XC5VLX110T Virtex-5 FPGA with a maximum clock frequency of 50 MHz. The values of signature generation and verification are 7.2 ms and 9.4 ms respectively.

The benchmark done in [26] implements ECDSA algorithm on an Intel Core 2 Duo processor with a speed of 1.83 GHz. The results show values of 2.88 ms and 8.53 ms for signature generation and verification times. In [27], different CPU devices have been benchmarked for ECDSA signature signing and verification. The WRT54GL is the most inefficient device that only performs 79 signature and 16 verification procedures per second. On the other hand, EEE PC 901 GO device has the best performance of 983 signature and 179 verification per second.

While there exists many benchmark studies to evaluate ECDSA based signature and verification times, there is a dearth of work that finds security processing delays based on the ITS standard complaint security algorithms and messages which is the main motivation behind this paper. Indeed, there has been very few papers which have addressed this important issue. In [28], PRESERVE security system in ITS has been implemented on FPGA and digital signature and verification delays were evaluated. The results showed that 400 signatures can be generated per second whereas only 30 signatures can be verified per second. However, no details about the processor speed and packet payload size has been provided. Similarly, work in [29] implemented ETSI ITS secured message and pointed out various complexities in the standard. Another work in [30] used values of 2 ms and 5 ms for digital signature and verification times respectively.

Nonetheless, as discussed before, there is no comprehensive work that benchmarks ITS standard compliant security packet overhead, signature and encryption times to the best of our knowledge. In this paper, We implement the ITS secured message and algorithms as per the standard using real experiments and test-bed to better assess their costs in terms of packets overheads and processing delays. Finally, we analyze the impact of ITS security compliant standards on the performance of V2X communications in realistic urban mobility scenarios using extensive simulations, in terms of achieved QoS, security levels, and safety awareness.

### 2.4. Performance Metrics

To evaluate the impact of ITS security on different applications, simulation based performance metrics are used. In literature, evaluation mechanism employs traditional wireless network performance indicators. These include packet delivery ratio measuring percentage of received packets, end-to-end-delay which computes the message latency and inter-arrival time that assesses time interval between arrival of two successive packets [17]. The authors in [31] have introduced a metric known as awareness quality level to find the impact of security on traffic awareness. In our previous work [17], we proposed novel safety awareness metrics that quantify the quantity of received messages as well as their accuracy. Moreover, to understand the effect of security, we proposed cryptographic packet loss metric that evaluates how many packets could not get verified before their time out.

The simulation based performance evaluation of ITS security standard has been done by [1]. The paper highlights the impact of security on packet delivery ratio and latency of safety messages in ITS. With an increase in vehicle density, the safety messages result in a reduced QoS. Another work in [32] has analyzed how security effects the vehicle awareness and suggest various certificate omission schemes to improve the awareness. As part of this paper, we use the standard compliant security benchmark values to analyze the impact on safety, QoS and security which has not been done in the previous works.

## 3. ITS Security Architecture

To efficiently manage the security features of ITS, both WAVE and ETSI standards have defined the security architecture. IEEE 1609.2 [14] in the WAVE standard and ETSI TS 103 097 [15] in the ETSI standard specifies the main security components, including the security headers, certificate format and security profiles. The security specifications in both standards are similar and suggest the use of Elliptic Curve Cryptography (ECC). Without loss of generality, the remainder of this section provides a high level overview of the security architecture as defined by the latest specifications of the ETSI TS 103 097 ITS standard, which was recently released to the public under the version 1.2.1 [15].

### 3.1. Security Architecture

**Figure 3:**
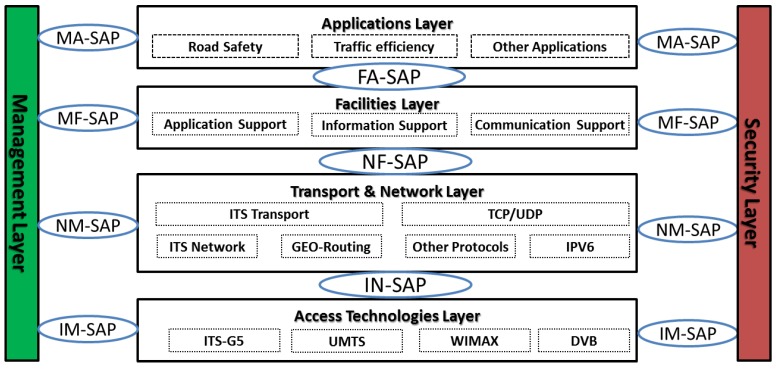
ETSI TC ITS reference architecture.

ETSI TC ITS has defined a reference architecture, as shown in Figure 3, which is quite similar to the US architecture [19]. It is based on a slightly modified IEEE 802.11p at the access layer, and enables new networking features based on geographical addressing at the network layer, and new facilities layer on top that enables a set of rich messages that support different types of applications. Compared to the US ITS Architecture, the ETSI TC ITS architecture includes more features at the network layer to support further communication scenarios, such as multi-hop forwarding. The facilities layer features are very similar in both architectures as most of them have been initially defined by the US standard (SAE) then adopted and slightly adapted by the EU standard (ETSI).

ETSI TC ITS defines security as a vertical layer adjacent to the access, networking and facilities layers, as shown in Figure 3. The corresponding security services are provided on a layer-by-layer basis through specific *Service Access Points* (SAP). In this context, ETSI TS 103 097 [15] specifies the main security components, including the security headers, certificate format and security profiles, and reuses as much as possible the existing IEEE 1609.2 security standard [14]. The remainder of this section provides a high level overview of these security components as defined by the latest specifications of ETSI TS 103 097, which was recently released to the public under the version 1.2.1 [15].

### 3.2. Security Headers

**Figure 4:**
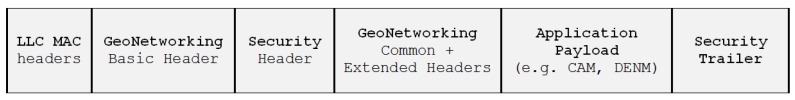
Secured GeoNetworking packet structure as defined in ETSI TC ITS.

ETSI TS 103 097 [15] defines different security headers and formats to ensure the interoperability of the different elements and security information that are being exchanged between the ITS stations for security purposes. The main security header is the *SecuredMessage* structure which specifies how to encode a generic security message, which is it self encapsulated inside a GeoNetworking packet, as shown in Figure 4.

As shown in Listing 1, a *SecuredMessage* starts with a 1-byte *protocol_version* field that should be incremented each time the security standard is updated. The current protocol version is 2 as defined by the latest ETSI TS 103 097 [15] specifications. Then, a variable-length vector *header_fields* is defined and which shall contain the different information required by the security layer [15], such as *generation_time* (0), *expiration* (2), *generation_location* (3), *its_aid* (5), *signer_info* (128), *encryption_parameters* (129), *recipient_info* (130), *etc.* Depending on the considered security profile, the sequence of these header fields should be encoded in ascending numerical order of their type values. Next, a message *payload* is included and which consist in a payload type (*i.e.*, *unsecured*, *signed*, *encrypted*, *signed_external*, or *signed_and_encrypted*) followed by a variable-length vector containing the actual message payload (e.g., CAM, DENM, *etc.*).

Listing 1: ETSI ITS SecuredMessage Structure.
Struct {
        uint8        protocol_version;
        HeaderField  header_fields<var>;
        Payload      payload_field;
        TrailerField trailer_fields<var>;
} SecuredMessage;        

According to the latest standard specifications, only one payload can be included in a *SecuredMessage*. Finally, a variable-length vector *trailer_fields* is encoded just after the payload. These trailer fields contain security information which is necessary to verify the messages integrity and authenticity using a signature. Depending on the considered security profile, the sequence of these trailer fields should be encoded in ascending numerical order of their type values. It should be noted that the exact content of a *SecuredMessage* is determined by the corresponding security profile (*cf.* Section 3.4) which will check its validity, prior to applying any security operations.

### 3.3. Certificate Format

ETSI TS 103 097 [15] proposes a new certificate format that specifies how to encode the different information required by each type of certificate (*i.e.*, Root certificate, AA certificate, *etc.*) as described in the ETSI ITS authority hierarchy [33].

As shown in Listing 2, an ETSI ITS Certificate starts with a 1-byte certificate *version* field that shall be set to 2 for conformance with the latest standard specifications [15]. The information of the certificate’s signer is then given by the *signer_info* field which can be of type *self* (0), *certificate_digest_with_sha256* (1), *certificate* (2), *certificate_chain* (3), or *certificate_digest_with_other _algorithm* (4). Next, the certificate’s subject name and type (e.g., root_ca, crl_signer, enrollment_credential, authorization_ticket, *etc.*) are given by the *subject_info* field; whereas the certificate’s subject attributes are given by the variable-length vector *subject_attributes*, such as *verification_key* (0), *encryption_key* (1), *assurance_level* (2), *its_aid_list* (32), *etc.*

Listing 2: ETSI ITS Certificate Structure.
Struct {
        uint8                version;
        SignerInfo           signer_info;
        SubjectInfo          subject_info;
        SubjectAttribute     subject_attributes<var>;
        ValidityRestriction  validity_restrictions<var>;
        Signature            signature;
} Certificate;

The certificate’s validity information is then encoded by the variable-length vector *validity_restrictions*, and whose elements can include the *time_end* (0), *time_start_and_end* (1), *time_start_and_duration* (2) and *region* (3). It should be noted that the elements of the *subject_attributes* and *validity_restrictions* fields should be encoded in ascending numerical order of their respective types. Finally, the signature field holds the certificate’s signature which is signed by the *Certificate Authority* (CA). This signature is calculated based of the preceding certificate fields.

### 3.4. Security Profiles

ETSI TS 103 097 [15] defines four main security profiles for CAMs, DENMs, generic messages and certificates. Each of the above security profiles defines the elements that should be part (or not) of a *SecuredMessage* as well as their order and number of occurrences. One of the main difference between the CAM and DENM security profiles consists in the content of the *signer_info* field. For DENM messages, *signer_info* should always include one element of type certificate; whereas, an element of type *certificate_digest_with_sha256* should be included in the normal case for CAM messages.

These security profiles are based on two main underlying security algorithms which will be discussed in the remainder of this section.

#### 3.4.1. Elliptic Curve Digital Signature Algorithm (ECDSA)

The first security algorithm is ECDSA [34] for signing and verifying exchanged messages, and whose objective is to ensure the integrity, authenticity and non-repudiation of the exchanged data. In this context, the ETSI TC ITS standard recommends the usage of the *ecdsa_nistp256_with_sha256* public key algorithm, even though the standard is flexible enough to support other algorithms, however, no further details are given [15].

**Figure 5:**
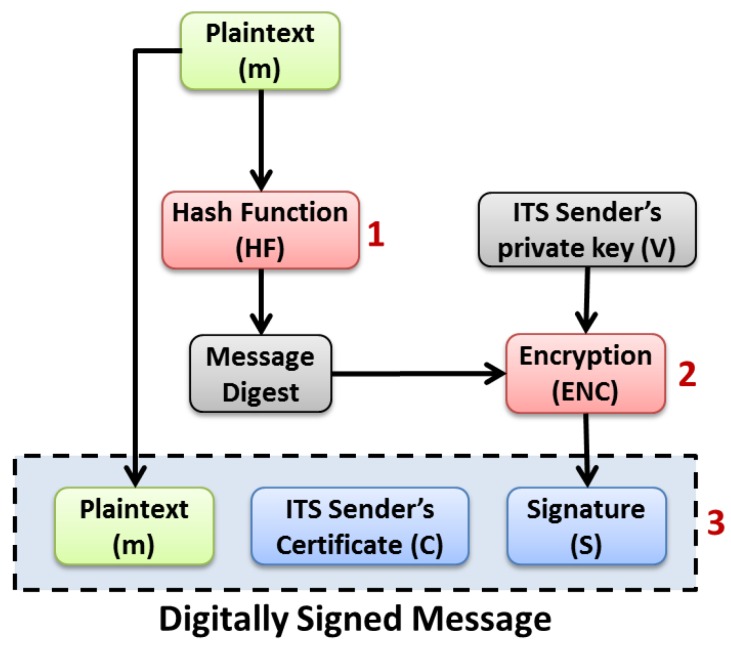
ECDSA based message signature process.

As shown in Figure 5, the message signature process works according to three main steps:

- A message digest is computed using a hash function (e.g., SHA-256) over the message plain text to be secured. For example, in case of a CAM message to be signed, the digest is computed over the CAM’s *protocol_version*, *header_fields*, *payload_field* and the length of the *trailer_fields* and the type of the signature trailer field;
- Then, the obtained message digest is encrypted using a public key algorithm (e.g., *ecdsa_nistp256_with_sha256*) and the private key of the ITS sender. The resulting encrypted message digest is known as the message’s signature;
- Finally, a digitally signed message is built based on the original message plain text, the computed signature and the ITS sender’s certificate. This secured message can thus be transmitted over the wireless channel.

It should be noted that, depending on the considered ETSI ITS security profile, the ITS sender’s certificate might not be included in every digitally signed message. For example, the element of type *certificate* is typically included in signed CAMs only periodically (every 1 s), or in case a *request_unrecognized_certificate* is received from a nearby ITS station. The main reason is to reduce the message overhead and improve the scalability of ITS communications.

**Figure 6:**
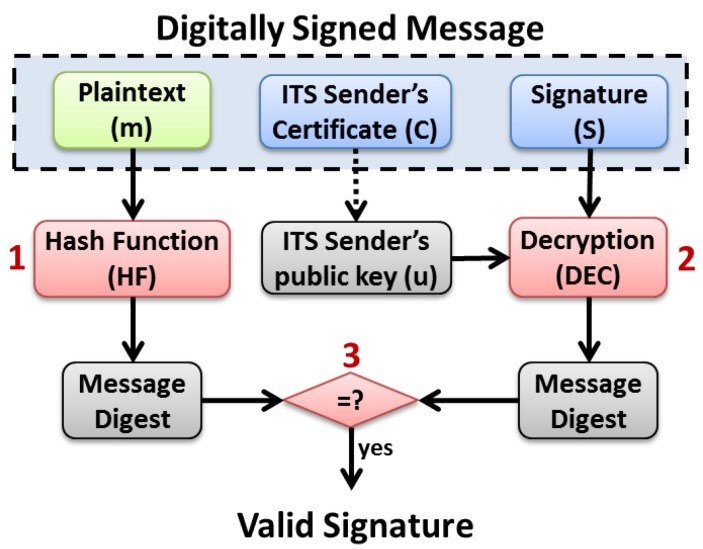
ECDSA based Message Verification Process.

As shown in Figure 6, once a signed message is received by an ITS station, the message verification process undergoes three main steps:

- The message plain text is extracted from the *SecuredMessage*, and a message digest is computed using a hash function (e.g., SHA-256).
- Then, the message’s signature is extracted from the *SecuredMessage*, and is decrypted using a public key algorithm (e.g., *ecdsa_nistp256_with_sha256*) and the sender’s public key (*i.e.*, contained in the received sender’s certificate). The obtained decrypted signature represents the message digest as originally computed by the sender.
- Finally, the receiver compares the decrypted message digest (step 2) with the newly computed message digest (step 1). If both digests are equal, the signature is thus considered as valid, as well as the integrity and authenticity of the received data.

It should be noted that, depending on the considered ETSI ITS security profile, the content of the *SecuredMessage* might also be checked against the rules of the security profile. For example, in case of the CAM security profile, the sender’s certificate is validated against timing, location and security considerations. In case the signer’s certificate is unknown, a *request_unrecognized_certificate* is generated.

#### 3.4.2. Elliptic Curve Integrated Encryption Scheme (ECIES) with Advanced Encryption Standard (AES)

The second security algorithm is ECIES with AES for encrypting and decrypting exchanged messages, and whose objective is to ensure the confidentiality of the exchanged data. The ECIES public key algorithm is used for the secure exchange of the AES secret key between the transmitter and receiver, while the ITS message is encrypted using the AES algorithm and the shared secret key. In this context, the ETSI TC ITS standard recommends the usage of the *ecies_nistp256* public key algorithm in addition to the *aes_128_ccm* symmetric algorithm (*i.e.*, Advanced Encryption Standard with a key size of 128 bits and the Counter with Cipher Block Chaining Message Authentication Code [35]). The standard is flexible enough to support other algorithms, however, no further details are given [15].

**Figure 7:**
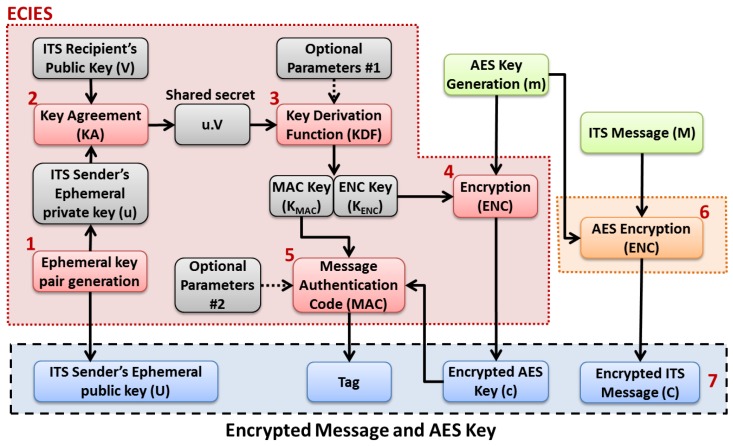
ECIES with AES based message encryption process.

As shown in Figure 7, the message encryption process undergoes seven main steps [36]:

- First, a pair of ephemeral key is generated, where the ephemeral private key is *u* and its public key is U=u·G, where *G* is the point of the considered elliptic curve, and “·” denotes a scalar multiplication;
- Second, a key agreement function (e.g., Diffie-Hellman with cofactor multiplication) is used to generated a shared secret using the sender’s ephemeral private key (*u*) and the receiver’s public key (*V*);
- Third, the obtained shared secret along with optional parameters (*i.e.*, null according to the ETSI TC ITS specifications) are fed into a key derivation function (KDF), based on KDF2 with SHA-256, whose output is the concatenation of the message authentication code (MAC) key ($K_{MAC}$) and the encryption key ($K_{ENC}$);
- Fourth, an AES secret key (m) is generated by the transmitter and is encrypted using a stream cipher and the encryption key $K_{ENC}$. The obtained encrypted AES key is denoted as *c*;
- Fifth, the encrypted AES key *c*, the MAC key $K_{MAC}$ and optional parameters (*i.e.*, null according to the ETSI TC ITS specifications) are fed into a MAC function with SHA-256 to generate a tag of 128 bits;
- Sixth, the ITS message (*M*) is encrypted using the AES algorithm (*i.e.*, *aes_128_ccm*) and the AES secret key which was generated in step 4;
- Finally, the sender’s ephemeral public key (*U*), the tag, the encrypted AES key (*c*) and the encrypted ITS message (*C*) are encapsulated into a secured message, along with other headers and fields, and which is transmitted through the wireless channel to the remote ITS receiver.

**Figure 8:**
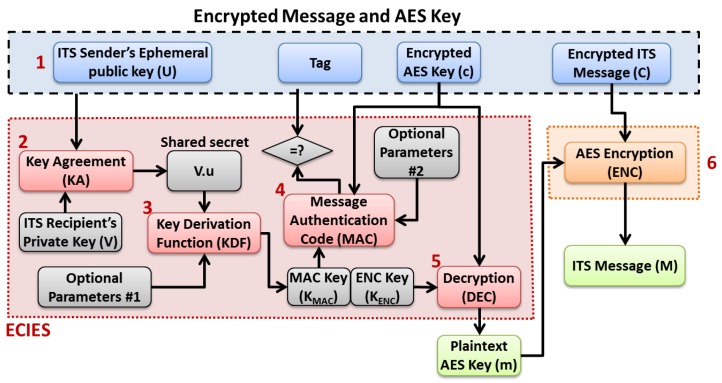
ECIES with AES based Message Decryption Process.

As shown in Figure 8, once an encrypted message is received by an ITS station, the message decryption process undergoes six main steps:

- First, the ITS sender’s ephemeral public key (*U*), the tag, the encrypted AES key (*c*) and the encrypted ITS message (*C*) are extracted from the received secured message;
- Second, the ITS sender’s ephemeral public key *U* and ITS receiver private key *V* are fed into a key agreement function to generate the shared secret V·u [36];
- Third, the shared secret along with optional parameters (*i.e.*, null according to the ETSI TC ITS specifications) are fed in a key derivation function (KDF), whose output is the concatenation of the message authentication code (MAC) key ($K_{MAC}$) and the encryption key ($K_{ENC}$);
- Fourth, the encrypted message *c*, the MAC key $K_{MAC}$ and optional parameters (*i.e.*, null according to the ETSI TC ITS specifications) are fed into a MAC function to generate a new tag that is compared with the received tag. If the values are different, the received secured message should be discarded by the receiver;
- Fifth, the received encrypted AES key *c* is decrypted using the encryption key ($K_{ENC}$), and the original AES key *m* is obtained;
- Finally, the encrypted ITS message *C* is decrypted using the AES key *m*, and the original ITS message *M* is obtained and can be further processed by the ITS communication stack and applications.

It should be noted that the step related to the secure exchange of the AES key between the transmitter and receiver (using ECIES) might not be done for every transmitted ITS message. Indeed, once an AES key is shared between both parties, subsequent ITS messages transmissions can be encrypted using the cached AES key (i.e. key store), reducing thus the overhead related to the ECIES public key algorithm. However, no further details are provided by the standard [15], for example, on how to maintain and manage such key stores, or on recommended AES keys cache retention period.

## 4. Test-Bed and Experimental Benchmark

In this section, we describe the test-bed setup to experimentally benchmark the performance of an ETSI ITS security layer implementation. While the addition of security mechanism improves application reliability, it comes with a computation cost. The three important security parameters that need to be considered in this regard include security overhead, ENCAP time (for security encapsulation of ITS packets) at the transmitter and DECAP time (for security decapsulation of ITS packets) at the receiver. Security overhead is the additional bytes that need to be added to the packet prior to its transmission. ENCAP procedure includes a step of signature and/or encryption. ENCAP time is the delay at the transmitter to prepare the secured message. DECAP procedure includes a step of verification and/or decryption. DECAP time is the delay at the receiver to verify and/or decrypt the packet.

### 4.1. Test-Bed Setup

**Figure 9:**
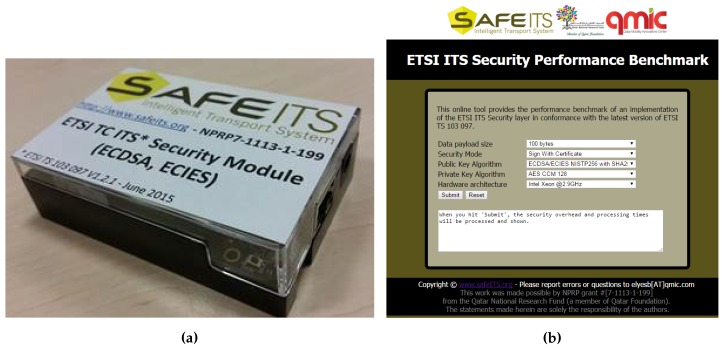
ETSI TC ITS Security Standard Compliant Experimental Test-Bed and Online Tool. (**a**) ETSI TC ITS Compliant Security Module (BeagleBone version); (**b**) Online tool for ITS Security Performance Benchmark [18].

To compute the security parameters based on the algorithms defined in the ITS standards, we perform experiments using a cryptography API on different hardware architectures [13]. The API known as Bouncy Castle allows the implementation of different cryptography procedures. The ETSI security header, certificate format and security profiles were implemented according to the latest standard specifications. ETSI TS 103 097 [15] was implemented as a standalone software module, as shown in Figure 9a, and was integrated with the GeoNetworking and Facilities layers through standard compliant Service Access Points [37]. In particular, the SN-ENCAP (signature and encryption) and SN-DECAP (verification and decryption) service primitives [37] were implemented to enable the security profiles (as discussed in Section 3). We also developed an online tool that provides all the security performance benchmark results as shown in Figure 9b. This tool is a useful calculator that can help researchers working in the ITS domain to compute realistic security overhead and processing delays for ITS messages using different processors.

An SN-ENCAP request [37] is sent from the networking and transport layer to the Security entity to request the encapsulation of an outbound message (e.g., CAMs *etc.*) into a SecuredMessage envelope according to a security profile. The outbound message is secured (e.g., signed and/or encrypted based on the considered security profile) and encapsulated into a SecuredMessage (as already described in Section 3) and is sent back to the requesting layer. The secured message is then encapsulated into a GeoNetworking packet which is transmitted over the wireless channel by the MAC layer (e.g., IEEE 802.11p). An SN-DECAP request [37] is sent from the networking and transport layer to the Security entity to decapsulate an inbound message from the SecuredMessage envelope. The message payload is extracted, verified and/or decrypted (based on the considered security profile) and made available to the networking and transport layer.

### 4.2. Experimental Results

For the sake of completeness of this study, different ECDSA and ECIES schema were implemented and benchmarked as listed in Table 2 for securing ITS communications (e.g., V2V, V2I).

We tested and validated our implementation during the 4th ETSI ITS Plugtest event and against the web validator named Fraunhofer FOKUS ETSI TS 103 097 [13]. Various security modes were benchmarked in terms of average security overhead, encap and decap operations per second (OPS) on three different CPU architectures as shown in Table 3. The selected processors include Intel i7, Intel Xeon and CubieBoard, as shown in Table 3. Encapsulation and decapsulation of ITS packets were performed in conformance with standard compliant security profiles [15] (*cf.* Section 3.4). The payload of ITS packet is taken as 100 bytes. The results were averaged over 1000 iterations within 95% confidence interval.

**Figure 10:**
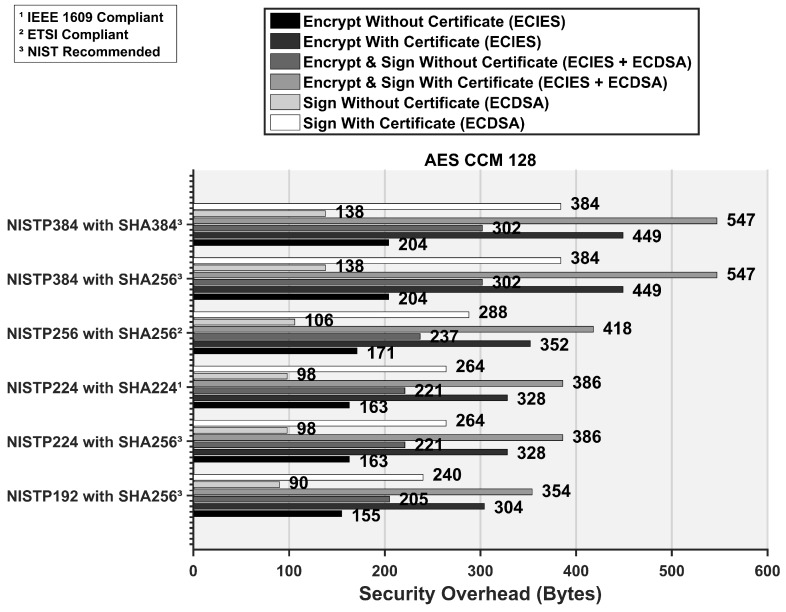
Security overhead required for different security procedures in conformance with the IEEE WAVE and the ETSI standard at AES CCM 128.

We display the required overhead in bytes for different security procedures at AES CCM 128 in Figure 10. Depending on the application requirements, a vehicle may choose to only sign or encrypt a packet or do the both with or without including its certificate in the secured message. As shown in Figure 10, the ETSI compliant security procedures can induce an additional security overhead of up to 418 bytes depending on the level of security. For the signature procedure, encryption procedure and combined signature & encryption procedure, addition of 106, 171 and 237 bytes are needed respectively. Inclusion of certificate further adds 180 bytes to the secured ITS packet.

**Figure 11:**
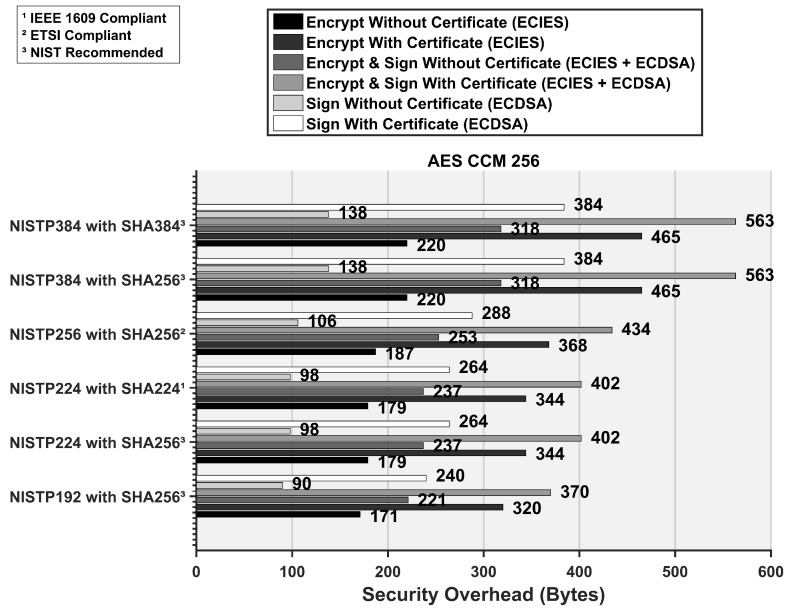
Security overhead required for different security procedures in conformance with the IEEE WAVE and the ETSI standard at AES CCM 256.

To present the security overhead values using AES CCM 256 security algorithm, we compile the results for such a setting in Figure 11. Since AES CCM value only effects the encryption scheme setting, the security overhead for signature procedure remains same. In comparison to the AES CCM 128 results, the packet overhead for procedures involving encryption increases by 15–20 bytes at AES CCM 256.

**Figure 12:**
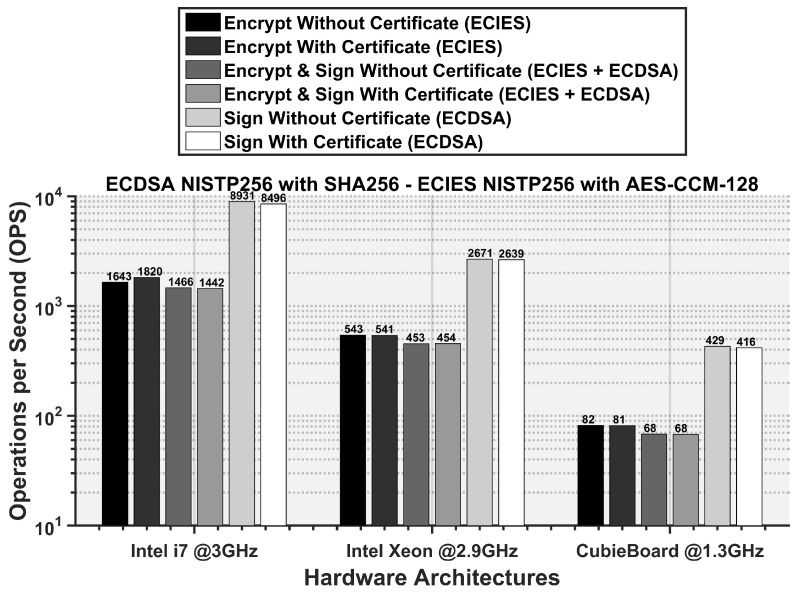
Required operations per second (OPS) for different ENCAP procedures in conformance with the ETSI standard.

In Figure 12, we show the achieved number of security operations per second (OPS) by the three hardware architectures for various ENCAP procedures. The ECDSA and ECIES algorithm parameters are set as per the ETSI ITS standard. Each of the security procedure has its own computational expense. The signature operation can be performed faster as compared to the encryption procedure. If the ITS packet only needs to be signed, an Intel Xeon processor can perform 2671 of these operations in a second. Similarly, for encryption procedure and combined signature & encryption procedures, 543 and 453 operations can be performed in one second. The addition of a certificate increases the packet overhead but does not add significant delay and hence, OPS are similar to the ones without certificate.

CPU architecture and processor speed is critical to the required OPS. It can be seen that the Cubie board offers the lowest ENCAP OPS of 81 if the packet is signed and encrypted along with a certificate. On the other hand, Intel i7 is most robust of three processors that can perform up to 1442 ENCAP OPS. Intel Xeon processor falls in the middle with ENCAP OPS of 453. Since ENCAP OPS is an indication of how fast a processor can perform security operation, a higher OPS performance shows benefit in terms of requiring less time to prepare a secured message. Hence, it reduces the packet transmission delay, decreasing the channel occupancy.

**Figure 13:**
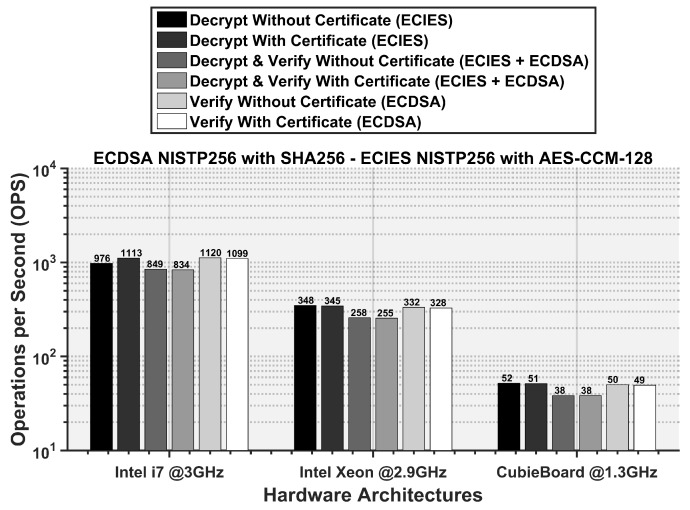
Required operations per second (OPS) for different DECAP procedures in conformance with the ETSI standard.

The DECAP operations per second are shown in Figure 13. A CubieBoard processor of 1.3 GHz can only perform 38–52 DECAP operations per second and hence, incurs a very high security processing delay at the receiver. In comparison, Intel Xeon processor with a speed of 2.9 GHz, is able to process 255–348 packets per second and a more powerful 3 GHz Intel i7 processor improves the DECAP rate to 1120 packets per second. The DECAP rate also depends on the level of security added to a packet. For example, if a packet is both signed and encrypted along with a certificate, it takes the highest time to process at the receiver. Therefore, such a security level should only be restricted to critical applications.

In Table 4, we display the ENCAP delay for different ECDSA and ECIES schema that are ETSI, IEEE WAVE and NIST compliant. Here we are considering the AES-CCM algorithm with 128 bits keys, while the other ECC based security algorithms (NISTP and SHA values) are varied as shown in Table 4. Since the use of lower NISTP and SHA values implies a lighter weight cryptography scheme, it results in lower ENCAP delay. For an Intel Xeon processor, the ENCAP delay required for encryption and signature without certificate procedure, is 1.13 ms, 1.54 ms and 6.12 ms for NISTP values of 192, 224, and 384 respectively.

We also present the DECAP delay for various cryptographic schema per ITS standards in Table 5. It can be seen that the security schemes with higher NISTP and SHA values results in large DECAP delays of up to 88ms. In terms of DECAP delays, Intel i7, Intel Xeon and CubieBoard processors are ranked from lower to higher delay values. Particularly, DECAP delay values for verify and decrypt with certificate procedure on an Intel Xeon processor requires 1.95 ms, 2.69 ms and 10.99 ms for NISTP values of 192, 224 and 384 respectively.

## 5. ITS Security Case Study in Smart cities

In this section, we present an ITS case study for smart cities to evaluate the impact of security on the application performance. We employ network and traffic simulations to perform this study and present detailed simulation setup and results in the next subsections.

### 5.1. Simulations Setup

We used NS-3 network simulator to model the ITS communications architecture. With the help of SUMO traffic simulator, we generated an urban setting of 2 km ×2 km road area. The average road length between two intersections is 500 m. The vehicle density is set to 200 vehicles/km^2^ representing a high density scenario. Each vehicle moves with a maximum speed of up to 80 km/h. We placed one infrastructure road side unit at every intersection *i.e.*, every 500 m to facilitate V2I communications. The simulation parameters are listed in Table 6.

To model the ITS applications of the smart cities, we implemented two applications. The first one is the safety application that requires exchange of periodic safety messages (*i.e.*, CAMs) between vehicles (v2V) whereas the second application is the query service such as optimal route guidance or online banking transaction that needs a request/reply between vehicles and the surrounding infrastructure (V2I). For the safety application, we used a payload size of 100 bytes. We add digital signature to each CAM packet and the security overhead calculated from Figure 10 is 106 bytes and 288 bytes without and with certificate respectively. Since CAM generation rate is 10 packets/second, the certificate is only sent once within this duration according to the ETSI standard (*cf.* Section 3.4).

We use the Intel Xeon and Intel i7 processors with a speed of 2.9 GHz and 3 GHz respectively for security processing. Medium and Fast Speed Security in this section refers to as the security processing using Intel Xeon processor and Intel i7 processors respectively. CubieBoard processor is not selected for simulations due to its slow processing speed which makes it unreasonable to be used by the ITS applications. The signature and verification duration for the safety application for medium and fast speed security is given in Table 6.

The query service application uses a packet size of 40 bytes plus the security overhead for the request sent from vehicle to infrastructure (up-link). The infrastructure processes the service and replies with a packet size of 200 bytes plus the security overhead back to the vehicle (down-link). We use encryption algorithm ECIES-NISTP 256-AES-CCM-128 for the query service application. The security overhead is 171 bytes whereas the encrypt and decrypt durations are listed in Table 6.

### 5.2. Simulations Results

**Figure 14:**
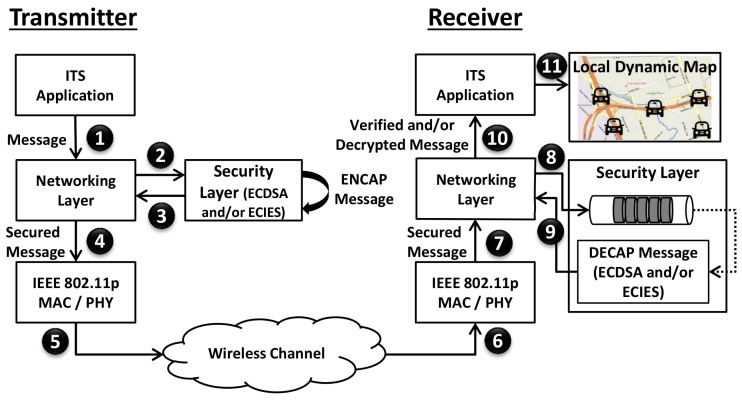
Secure ITS message transmission.

We present the simulation results in this subsection categorized into safety application and query application. The secure ITS message transmission procedure is shown in Figure 14. Each ITS packet is first signed and/or encrypted at the transmitter before passing it to the lower layers. Using the wireless channel, packet is transmitted to the receiver. At the receiver, packet is verified and/or decrypted. The verified packets are finally passed to the application layer and local dynamic map is evaluated.

#### 5.2.1. Safety Application

For the safety application, it is critical to analyze the performance in terms of QoS, security and safety awareness of vehicles. To achieve this task, we demonstrate the key metrics as follows.

##### **QoS Metrics** 

**Figure 15:**
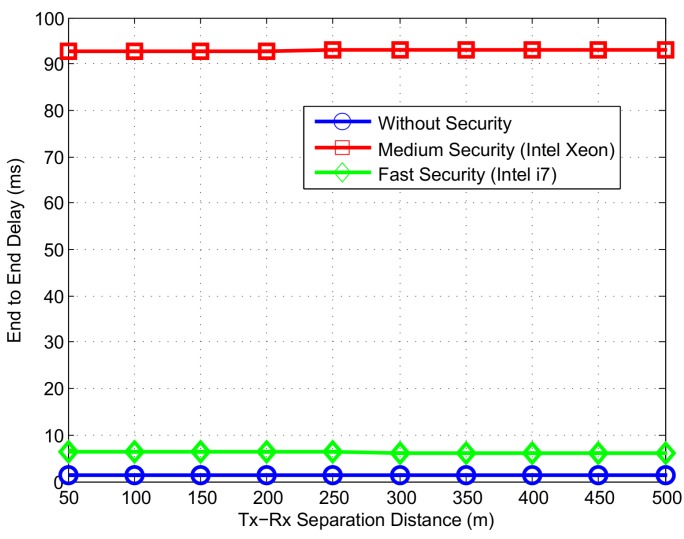
End-to-End Delay at different levels of security.

We show the end-to-end delay of CAMs for the simulated scenario in Figure 15. End-to-End Delay is defined as the difference between steps 10 (when packet is passed to the application layer at the receiver) and 1 (when packet is generated at the transmitter) in Figure 14. Without a security mechanism, the end-to-end delay remains below 2 ms at different Tx-Rx separation distances. When ECDSA-256-SHA-256 algorithm is used for digital signature of CAMs, end-to-end delay rises due to additional packet overhead and security computation/processing delay. Using medium speed security, the end-to-end delay goes up to 90 ms while a faster processor results in an end-to-end delay of up to 8 ms. Since fast speed security requires less than 1ms time for verify procedure, it does not cause congestion of the security queue at the receiver, resulting in a end-to-end delay closer to the case when no security is used. Finally, it should be noted that the average end-to-end delay is not impacted by the Tx-Rx separation distances due to the fact that propagation delay is quite negligible in comparison to the security processing delay.

**Figure 16:**
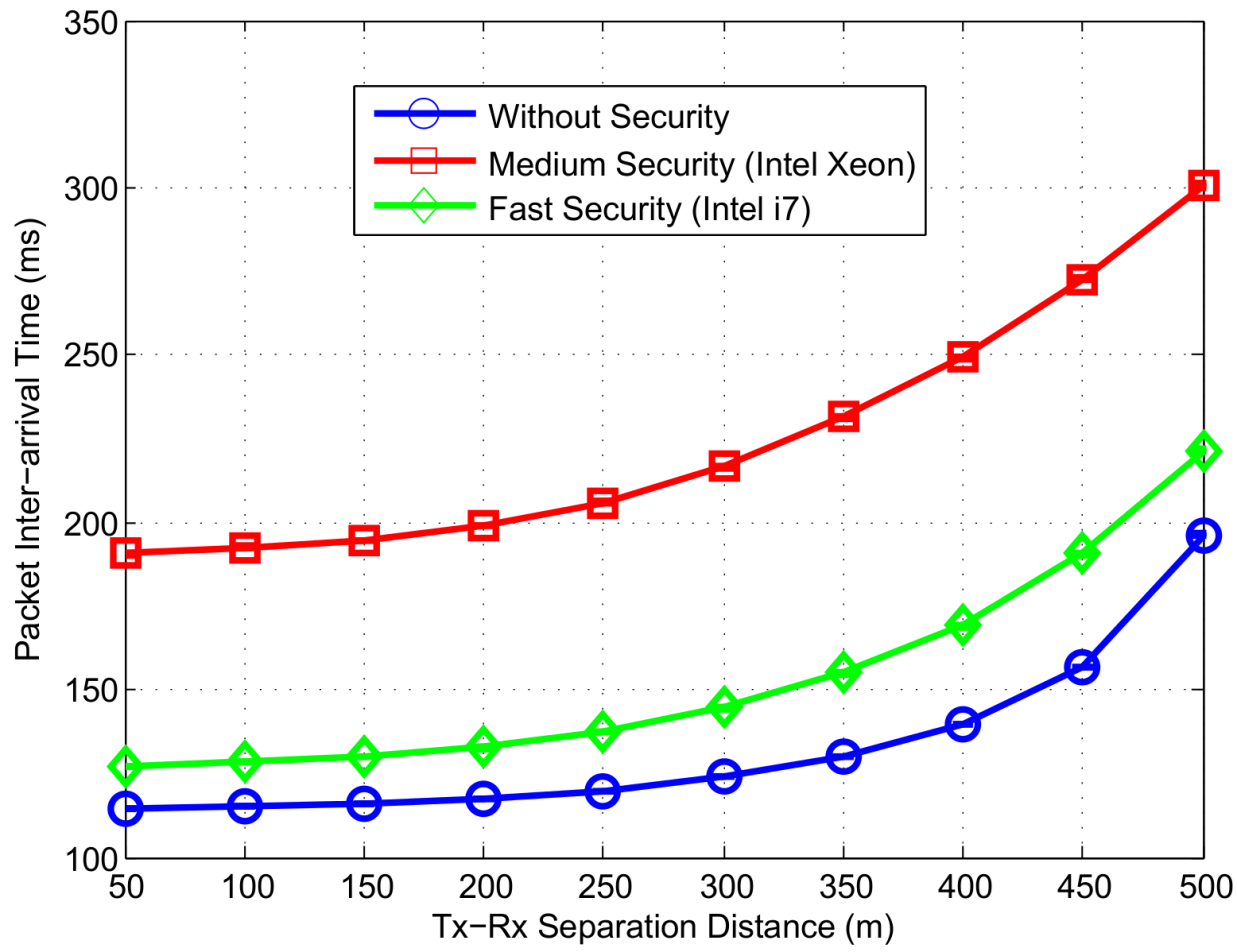
Packet Inter-arrival time at different levels of security.

To evaluate the time between arrival of two consecutive CAMs from the same sender, we display packet inter-arrival time in Figure 16. Since CAMs are transmitted every 100 ms, an inter-arrival time closer to this value is deemed ideal. Indeed, the packet inter-arrival time is less than 200 ms when security procedure is not used. The increase in inter-arrival time from 100 ms value is due to packet loss due to collisions and fading. Using security signature algorithm, the inter-arrival time goes up to 300 ms and 220 ms for the medium and fast speed security respectively.

**Figure 17:**
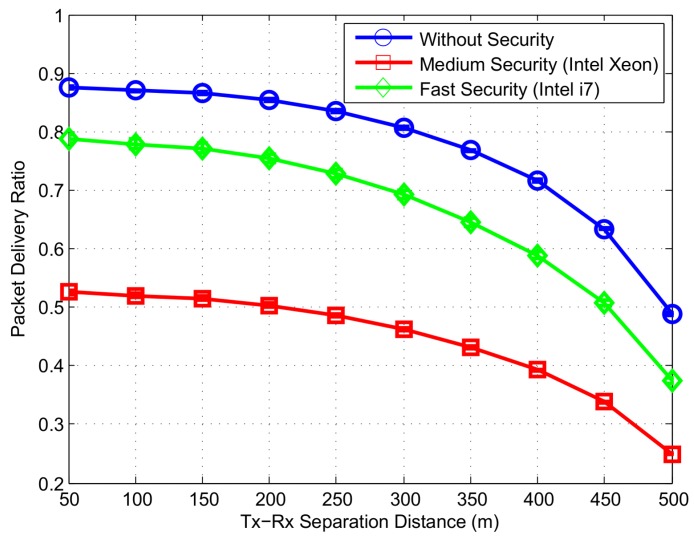
Packet Delivery Ratio (PDR) at different levels of security.

We display the packet delivery ratio (PDR) of CAM in Figure 17. The results show that the PDR for safety messages without security procedure remains up to 0.8 within a Tx-Rx distance of 300 m. The resulting loss is due to collisions caused by high number of vehicles sending messages simultaneously. Another reason of packet loss is the reduction in received signal strength due to fading. When the security procedure is added, the PDR further reduces to increased packet overhead and security processing delay. For the medium speed security, the PDR is equal to 0.48 and it increases by 0.22 if high speed security is used.

##### **Security Metrics** 

**Figure 18:**
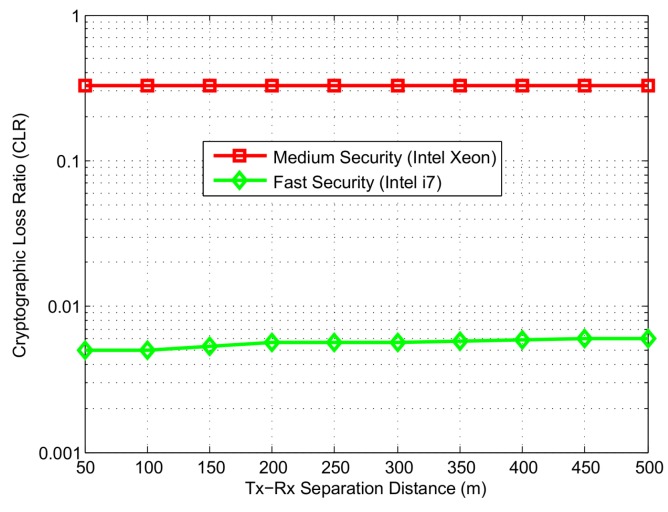
Cryptographic Loss Ratio (CLR) at different levels of security (Logarithm scale at *y*-axis).

We display the impact of security on safety messages in Figure 18. Since security mechanism increases the packet overhead that raises the channel occupancy time, hence it requires more time to transmit the packet. Additionally, it requires certain time to verify the packet depending on the processor speed. This causes the safety messages to wait in the queue for their turn to get verified. CAMs that could not get verified within a time out of 100ms are dropped from the queue and resulting loss is known as cryptographic loss. We define cryptographic loss ratio (CLR) as the ratio of number of packets that get dropped due to timeout to the number of packets that arrived at the queue for verification. As shown in Figure 18, CLR of CAMs is 0.33 depending on the Tx-Rx separation distance. As per intuition, the security speed is inversely proportional to the CLR. For the fast security speed, packets arriving at the receiver security queue do not need to wait much, hence CLR is less than 0.01.

##### **Safety Awareness Metrics** 

**Figure 19:**
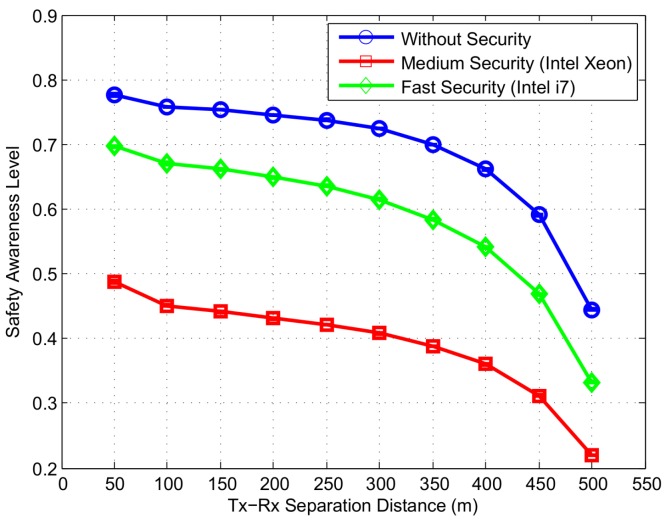
Normalized Weighted Error based Safety Awareness Level metric ($\mu \hat{SAL}$) at different levels of security.

To evaluate the safety awareness of vehicles as part of the safety applications, we display the normalized weighted error based safety awareness level metric ($\mu \hat{SAL}$) proposed in our previous work [17]. The proposed metric uses percentage of received information and its accuracy in vehicle’s LDM to compute the level of safety awareness. The resulting metric which has a value between 0 and 1 indicates the fidelity of vehicle’s LDM and hence, applications reliability. Figure 19 displays the $\mu \hat{SAL}$ metric depending on the level of security used. Without security mechanism, $\mu \hat{SAL}$ metric has a value of 0.72 within 300 m distance. The graph further shows the safety awareness for the two processors used in simulations. Medium speed security results in a safety awareness of 0.4 within a distance of 300 m which increases to 0.62 when fast speed security is used.

#### 5.2.2. Query Application

The query application utilizes a request-reply based data generation model. We randomly select vehicles on the road that send a query request service to the nearest infrastructure node. Upon receiving the request message, the infrastructure node processes the query and sends a reply back to the vehicle. Only 10%–20% of the vehicles generate query messages per second. Each of the query message is encrypted using ECIES-NISTP256-AES-CCM-128 algorithm. We display the results related to query application in Table 7. Without security, the success rate is 75%. With medium speed security used for encryption and decryption, success rate is reduced to 56%. On the other hand, fast speed security mechanism results in success rate of 57%.

Unlike safety messages, query messages do not have a specific time out after which packet is dropped from the queue. Therefore, the end-to-end delay for this application is around 4 ms in the absence of security mechanism. Addition of security results in an increased delay of 13.2–195.4 ms in the delay depending on the processor speed.

### 5.3. Lessons Learned and Open Challenges

The benchmark values of packet overhead and processing delays presented in this paper provide an insight on computation cost associated with the security procedure in ITS applications. The computation cost is dependent on the level of security added to the application *i.e.*, inclusion of signature, encryption and certificate. An application that requires ITS packets to be signed and encrypted along with a certificate results in highest packet overhead, thus impacting the channel occupancy. The other factor that dictates the security computation cost is the processor speed. A more robust processor results in shorter time to handle the security procedure at transmitter and receiver sides.

Based on our simulation studies, we infer the impact of security on two different ITS applications. For the safety applications, digital signature mechanism is used to ensure message reliability. A medium speed security results in a significant decrease in QoS. Particularly, the inter-arrival delay is increased up to 60 ms and and packet delivery ratio is reduced by 30% as compared to a faster speed security. A similar trend is seen in the cryptographic loss ratio which is increased by 34% when a lower speed processor is used. Finally, the faster of the two processors provides a 20% higher safety awareness level.

For the query based applications, we use the encryption algorithm to achieve data security. Since these non-safety applications don’t suffer from cryptographic loss (due to no timeout value as in the case of safety applications), the packet success rate is not much effected by the processor speed. However, the medium speed security results in an increased end-to-end delay of more than 180 ms which is significant in the context of some non-safety applications.

Based on the results, we can clearly deduce that the ITS applications require a fast speed security for efficient exchange of secured messages. For this purpose, specialized hardware security module (HSM) or trusted platform module (TPM) may be utilized to accelerate the cryptographic operations by using higher CPU frequencies. One drawback of such modules is that their cost could be higher than an entry level vehicle. Moreover, further studies are needed to assess the additional cost of such modules against the QoS benefit in the context of ITS based smart cities.

Another solution to improve the ITS application QoS while maintaining the required security level is the use of adaptive security frameworks that are able to dynamically tune the security parameters at run time based on context and application requirements. One such technique proposed in [38] uses prioritized security verification of ITS messages from nearby vehicles that cause a greater safety concern, hence improving safety awareness. Decentralized congestion control is another method that can enhance the application QoS by adapting the wireless transmission parameters according to the channel load [39].

Finally, the use of traffic social networking could greatly improve the reliability of ITS based smart cities [40]. Vehicle could develop a trust based reputation level mechanism based on their interaction with the neighborhood. Vehicles that have a history of secured data exchange could afford to reduce the security levels for future communications. Similarly, vehicles with a high social interaction are less expected to be malicious and can act as data forwarders for ITS communications.

## 6. Conclusions

In this paper, we discuss the Elliptic Curve Cryptography (ECC) based algorithms for the ITS applications of smart cities. As part of the current ITS standards, these algorithms provide digital signature, certification and encryption mechanism services for the ITS messages. Using an experimental test-bed, we implement the ECC based signature and encryption procedures, and benchmark their performance in terms of security packet overhead and security processing delays. We also develop an online tool that provides the detailed security benchmark results based on our experimental study. We further implement an ITS application scenario using NS-3 and SUMO simulations. Based on benchmarked security parameter values, we analyze their impact on QoS, security and safety of ITS applications. Finally, we highlight the important lessons learned and present future research challenges to improve the security of ITS communications.

## Figures and Tables

**Table 1:** Security attacks, compromised security requirements and countermeasures.

Security Attack	Compromised Security Requirement	Countermeasure
Denial of Service (DoS)	Availability	Digital Signature
Jamming, Flooding	Availability	Digital Signature
Sybil	Availability, Authentication	Digital Signature
Malware, Spamming, Black hole, Grey hole, Sink hole, Warm hole	Availability, Authentication	Digital Signature
Eavesdropping	Confidentiality	Encryption
Data Interception	Confidentiality	Encryption
Falsified Entities	Authentication, Authorization	Digital Signature and Encryption
Cryptographic Replication	Authentication, Authorization	Digital Signature and Encryption
GNSS Spoofing	Authentication, Authorization	Digital Signature and Encryption
Timing	Authentication, Authorization	Digital Signature and Encryption
Masquerading	Data Integrity	Digital Signature with Certificate
Data Playback	Data Integrity	Digital Signature with Certificate
Data Alteration	Data Integrity	Digital Signature with Certificate

**Table 2:** Benchmarked security algorithms.

Security	ECDSA for Signing and Verifying ITS Safety Messages (e.g., CAMs)	ECIES with AES for Encrypting and Decrypting Sensitive ITS Communications
ETSI TC ITS	ECDSA NISTP-256 with SHA-256	ECIES NISTP-256 with AES CCM 128/256 *
IEEE 1609.2	ECDSA NISTP-224 with SHA-224	ECIES NISTP-224 with AES CCM 128/256 *
NIST	ECDSA NISTP-192 with SHA-256	ECIES NISTP-192 with AES CCM 128/256 *
	ECDSA NISTP-224 with SHA-256	ECIES NISTP-224 with AES CCM 128/256 *
	ECDSA NISTP-384 with SHA-384	ECIES NISTP-384 with AES CCM 128/256 *

* AES CCM 128 is the recommended private key algorithm by ITS standards.

**Table 3:** Benchmarked hardware architectures.

Hardware Architecture (CPU)	Description
Intel i7 (Fast Speed)	Intel(R) Core(TM) i7-4610 M CPU @ 3.00 GHz - 64 bits OS
Intel Xeon (Medium Speed)	Intel(R) Xeon(R) E5-2667 CPU @ 2.90 GHz - 64 bits OS
CubieBoard (Slow Speed)	ARM(R) Cortex-A8 AM335x CPU @ 1 GHz - 32 bits OS

**Table 4:** ENCAP Delay in milliseconds (ms) for different cryptographic schema on CubieBoard (CB), Intel Xeon (Xn) and Intel i7 (i7) processors, using the AES-CCM algorithm with 128 bits keys.

Security Level	NISTP192-SHA256			NISTP224-SHA256			NISTP224-SHA224			NISTP384-SHA256			NISTP384-SHA384		
	CB	Xn	i7	CB	Xn	i7	CB	Xn	i7	CB	Xn	i7	CB	Xn	i7
Sign w/o Certificate ^a^	1.39	0.19	0.06	1.77	0.26	0.12	1.76	0.26	0.16	6.32	0.85	0.36	6.21	0.85	0.39
Sign with Certificate ^a^	1.44	0.20	0.07	1.83	0.27	0.08	1.81	0.27	0.09	6.39	0.86	0.42	6.31	0.86	0.38
Encrypt w/o Certificate ^b^	6.57	0.92	0.29	8.58	1.28	0.42	8.65	1.27	0.40	42.88	5.25	1.79	42.82	5.29	1.79
Encrypt with Certificate ^b^	6.58	0.93	0.30	8.62	1.29	0.40	8.68	1.28	1.18	42.93	5.29	1.78	42.87	5.30	1.92
Sign & Encrypt w/o Certificate ^c^	8.01	1.13	0.38	10.42	1.54	0.51	10.47	1.54	0.60	49.35	6.12	2.10	49.21	6.12	2.21
Sign & Encrypt with Certificate ^c^	8.06	1.14	0.39	10.55	1.56	0.78	10.54	1.55	0.53	49.43	6.17	2.11	49.29	6.14	2.12

^a^ Messages are digitally signed using ECDSA; ^b^ Messages are encrypted using ECIES with AES; ^c^ Messages are encrypted using ECIES with AES and digitally signed using ECDSA.

**Table 5:** DECAP Delay in milliseconds (ms) for different cryptographic schema on CubieBoard (CB), Intel Xeon (Xn) and Intel i7 (i7) processors, using the AES-CCM algorithm with 128 bits keys.

Security Level	NISTP192-SHA256			NISTP224-SHA256			NISTP224-SHA224			NISTP384-SHA256			NISTP384-SHA384		
	CB	Xn	i7	CB	Xn	i7	CB	Xn	i7	CB	Xn	i7	CB	Xn	i7
Verify w/o Certificate ^a^	10.71	1.49	0.47	13.98	2.08	0.69	14.26	2.06	1.04	66.79	8.35	3.46	67.31	8.38	3.63
Verify with Certificate ^a^	10.88	1.52	0.48	14.03	2.10	0.67	14.55	2.07	0.78	66.84	8.34	3.33	67.55	8.46	3.06
Decrypt w/o Certificate ^b^	10.39	1.44	0.48	13.50	1.99	0.65	13.82	1.97	0.75	64.26	7.93	2.73	64.86	8.03	2.75
Decrypt with Certificate ^b^	10.65	1.45	0.49	13.69	1.99	0.67	14.02	1.99	1.53	64.61	7.99	2.74	65.24	8.08	2.83
Verify & Decrypt w/o Certificate ^c^	14.00	1.93	0.64	18.24	2.71	0.88	18.64	2.69	1.55	86.41	10.78	3.67	87.44	10.87	3.77
Verify & Decrypt with Certificate ^c^	14.31	1.95	0.66	18.58	2.69	1.48	18.92	2.70	1.04	87.06	10.82	3.68	87.78	10.99	3.71

^a^ Messages are digitally verified using ECDSA; ^b^ Messages are decrypted using ECIES with AES; ^c^ Messages are digitally verified using ECDSA and decrypted using ECIES with AES.

**Table 6:** Simulation parameters.

Parameter		Value
Traffic	Road Area	2 km ×2 km
	No. of Lanes	6 (3 lanes per direction)
	Vehicle Density	200 vehicles/km^2^
	Vehicle Speed	80 km/h
CAM (Safety Application)	Payload Size	100 bytes
	Security Algorithm	ECDSA-256-SHA-256
	Security overhead	106 bytes (Sign without certificate)
		288 bytes (Sign with certificate)
	Generation Interval	100 ms
	Data Rate	6 Mbps
	Transmission Range	500 m
	Sign Duration	0.37 ms (Medium Speed)
		0.11 ms (Fast Speed)
	Verify Duration	3.01 ms (Medium Speed)
		0.89 ms (Fast Speed)
Query Application	Payload Size	40 bytes (Uplink)
		200 bytes (Downlink)
	Security Algorithm	ECIES-NISTP256 AES-CCM-128
	Security overhead	171 bytes (Encrypt without certificate)
	Data Rate	6 Mbps
	Transmission Range	500 m
	Encrypt Duration	1.84 ms (Medium Speed)
		0.61 ms (Fast Speed)
	Decrypt Duration	2.87 ms (Medium Speed)
		1.02 ms (Fast Speed)
Propagation Model	Pathloss	Dual slope
	Fading	Nakagami *m* = 1, 1.5, 3

**Table 7:** Query Application Performance.

	No. of Transmitted Requests	No. of Received Requests	No. of Received Replies	Success Rate (Request-Reply)	End-to-End Delay (Request-Reply)
Without Security	37.8	31.2	28.17	75%	4 ms
Medium Security (Intel Xeon)	37.8	28.1	21.41	56%	195.4 ms
Fast Security (Intel i7)	37.8	28.45	21.48	57%	13.2 ms
